# Signature Construction Associated with Tumor-Infiltrating Macrophages Identifies IRF8 as a Novel Biomarker for Immunotherapy in Advanced Gastric Cancer

**DOI:** 10.3390/ijms26031089

**Published:** 2025-01-27

**Authors:** Wanqian Liao, Yu Wang, Rui Wang, Bibo Fu, Xiangfu Chen, Ying Ouyang, Bing Bai, Ying Jin, Yunxin Lu, Furong Liu, Yang Zhang, Dongni Shi, Dongsheng Zhang

**Affiliations:** 1 Department of Medical Oncology, State Key Laboratory of Oncology in South China, Collaborative Innovation Center for Cancer Medicine, Sun Yat-sen University Cancer Center, Guangzhou 510060, China; liaowq@sysucc.org.cn (W.L.); wangyu@sysucc.org.cn (Y.W.); fubb@sysucc.org.cn (B.F.); baibing@sysucc.org.cn (B.B.); jinying1@sysucc.org.cn (Y.J.); luyx@sysucc.org.cn (Y.L.); liufr@sysucc.org.cn (F.L.); zhangyang@sysucc.org.cn (Y.Z.); 2 Integrated Traditional Chinese and Western Medicine Research Center, Sun Yat-sen University Cancer Center, Guangzhou 510060, China; wangrui@sysucc.org.cn (R.W.); chenxf@sysucc.org.cn (X.C.); ouyangy1@sysucc.org.cn (Y.O.); 3 Department of Experimental Research, State Key Laboratory of Oncology in South China, Collaborative Innovation Center for Cancer Medicine, Sun Yat-sen University Cancer Center, Guangzhou 510060, China

**Keywords:** AGC, TAMs, prognostic signature, immunotherapy prediction, *IRF8*

## Abstract

Advanced gastric cancer (AGC) is characterized by poor prognosis and limited responsiveness to immunotherapy. Tumor-associated macrophages (TAMs) play a pivotal role in cancer progression and therapeutic outcomes. In this study, we developed a novel gene signature associated with M1-like TAMs using data from the Gene Expression Omnibus (GEO) and The Cancer Genome Atlas (TCGA) to predict prognosis and immunotherapy response. This gene signature was determined as an independent prognostic indicator for AGC, with high-risk patients exhibiting an immunosuppressive tumor immune microenvironment (TIME) and poorer survival outcomes. Furthermore, Interferon regulatory factor 8 (*IRF8*) was identified as a key gene and validated through in vitro and in vivo experiments. *IRF8* overexpression reshaped the suppressive TIME, leading to an increased presence of M1-like TAMs, IFN-γ^+^ CD8^+^ T cells, and Granzyme B^+^ CD8^+^ T cells. Notably, the combination of *IRF8* overexpression and anti-PD-1 therapy significantly inhibited tumor growth in syngeneic mouse models. AGC patients with elevated *IRF8* expression were found to be more responsive to anti-PD-1 treatment. These findings highlight potential biomarkers for prognostic evaluation and immunotherapy in AGC, offering insights that could guide personalized treatment strategies.

## 1. Introduction

Gastric cancer (GC) is a highly aggressive malignancy with significant global morbidity and mortality [1]. Especially for AGC, the prognosis remains poor due to limited treatment options [2]. Thus, new strategies are urgently needed to recognize high-risk AGC patients.

In recent years, immunotherapy has shown promising clinical benefits across various advanced cancers [3]. However, GC is a highly heterogeneous disease with considerable variability in its response to immunotherapy [4]. This variability underscores the need to further explore the mechanisms driving immunotherapy resistance in some AGC patients.

The TIME is composed of diverse cell types and extracellular components. Growing evidence suggests that the dynamic evolution of TIME significantly impacts GC progression and therapeutic resistance [5,6]. TAMs are the main component of tumor-infiltrating leukocytes, exhibiting a high degree of plasticity. TAMs can be recruited and polarized into either M1 or M2 phenotypes in response to various cytokines, chemokines, and other signals within the TIME [7,8]. Generally, pro-inflammatory M1-like TAMs exert anti-tumor effects and reduce treatment resistance, whereas anti-inflammatory M2-like TAMs facilitate immune evasion and tumor progression [9]. A higher M1/M2 ratio is associated with an immune-active state and favorable prognosis [10,11,12]. High expression of M1-like TAM-related biomarkers has been shown to significantly inhibit the malignant behavior of osteosarcoma, highlighting its essential role in suppressing tumor progression [13]. Moreover, preclinical studies have demonstrated that targeting specific molecules can effectively induce the polarization of TAMs toward the M1 phenotype, thereby enhancing the therapeutic efficacy of PD-1 immune checkpoint inhibitors [14]. This mechanism illustrates the functional advantages of M1-like TAMs and provides new directions for tumor immunotherapy. These findings emphasize the potential of reprogramming TAMs toward the M1 phenotype as a promising approach to strengthen anti-tumor immunity and improve clinical outcomes [15,16]. Therefore, a deeper understanding of the crosstalk between GC and TAMs could enhance tumor immunity [17].

In this study, our bioinformatic analysis revealed that high infiltration of M1-like TAMs was positively correlated with improved survival in AGC patients. As a result, we constructed a novel prognostic model linked to M1-like TAMs for AGC. Furthermore, we selected *IRF8* as a key gene within the model and performed in vivo and in vitro experiments to confirm its role in macrophage polarization and immune activation. This prognostic signature offers new insights into predicting prognosis and immune evasion in AGC.

## 2. Results

### 2.1. Identification of M1-like TAM-Related Subgroups

**Figure 1:**
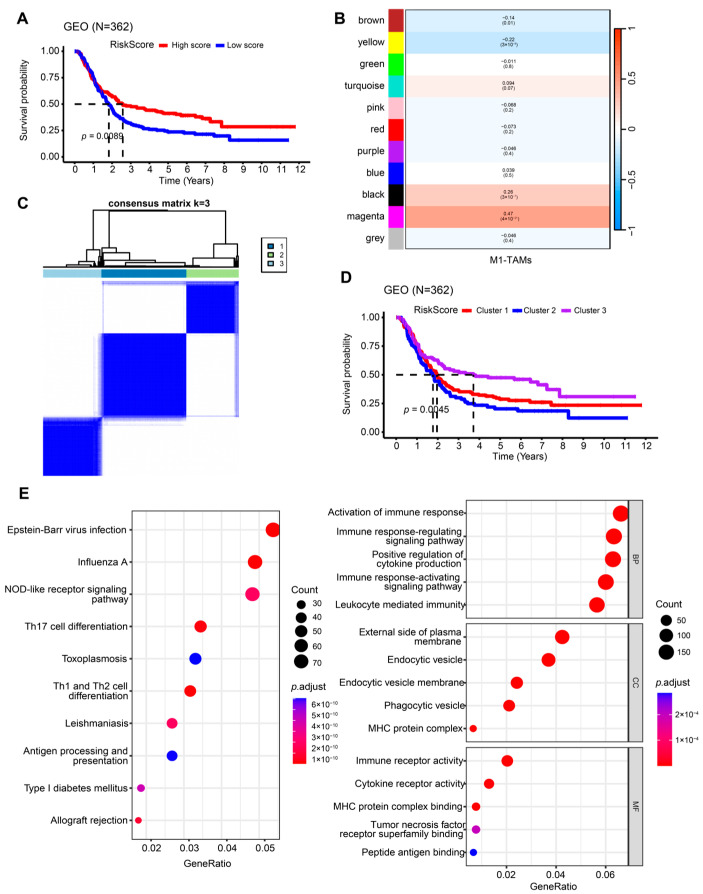
Identification of M1-like TAM-related clusters. (**A**) Survival analysis of the patients with high and low infiltration of M1-like TAMs grouped by the median CIBERSORT-based M1 macrophage scores. (**B**) WGCNA identified an M1-like TAM-related module. (**C**) The heatmap of the consensus matrix showing the optimal values for clusters was K = 3. (**D**) The KM curve revealed the difference in OS of the three clusters. (**E**) Differential gene GO and KEGG enrichment analysis. GO, Gene Ontology; KEGG, Kyoto Encyclopedia of Genes and Genomes; KM, Kaplan–Meier; TAMs, tumor-associated macrophages; WGCNA, weighted correlation network analysis.

The study workflow is outlined in Figure S1. We enrolled 362 AGC patients from the combined cohorts (GSE66229, GSE57303, GSE15459) with a complete gene expression matrix and clinical information in the analysis. Using the CIBERSORT algorithm, we quantified immune cell infiltration within the TIME. We found that patients with lower infiltration of M1-like TAMs showed a worse prognosis (log-rank *p* = 0.0089, Figure 1A). Next, weighted correlation network analysis (WGCNA) was performed to construct a gene co-expression network and determine the critical module associated with M1-like TAM infiltration. The magenta module showed the highest correlation by setting the soft threshold to 4 (cor = 0.47, *p* = 4 × 10^−21^) (Figure 1B and Figure S2A,C).

We then carried out a univariate Cox regression analysis to yield 36 genes correlated with overall survival (OS) among the magenta module. Unsupervised consensus cluster analysis based on these genes was conducted to divide the subjects into three subgroups (Figure 1C and Figure S2D–F). As shown in Kaplan–Meier (KM) curves, patients in Cluster 2 had worse OS, while those in Cluster 3 exhibited the greatest outcomes (log-rank *p* = 0.0045, Figure 1D).

We identified 3320 differentially expressed genes (DEGs) in three subgroups using the limma package to explore the underlying mechanism leading to different prognoses. Further, we selected 753 prognosis-related DEGs through univariate Cox regression analysis for functional enrichment analyses. Gene Ontology (GO) and the Kyoto Encyclopedia of Genes and Genomes (KEGG) revealed that the DEGs were mainly enriched in antigen processing and presentation, Th1 and Th2 differentiation, and regulation of immune response (Figure 1E). Overall, the results confirmed a significant prognostic difference among the M1-like TAM-related subgroups, which may be explained by the regulation of immune-associated pathways.

### 2.2. Construction and Validation of the M1-like TAM-Related Risk Signature for Independent Prognostic Prediction

**Figure 2:**
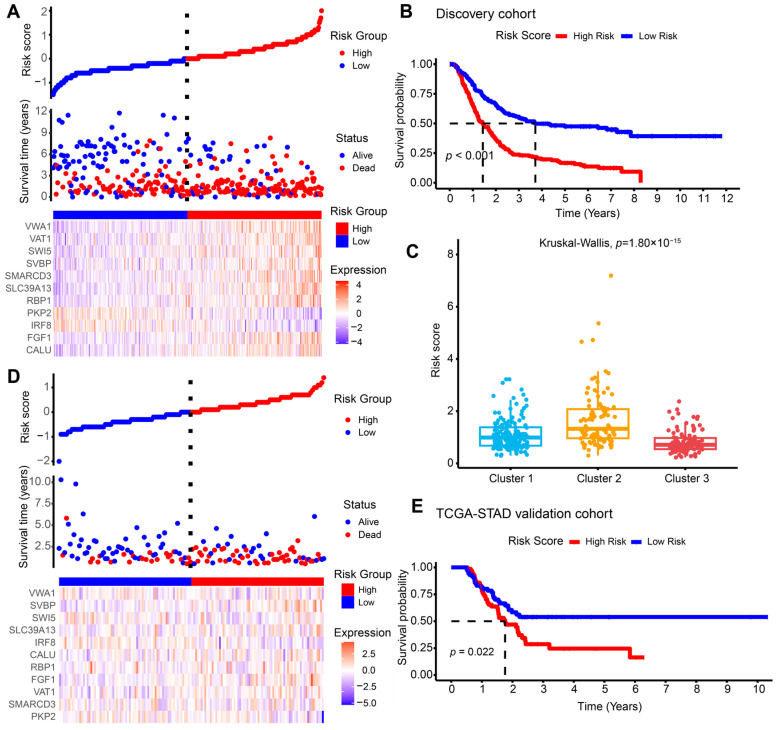
Construction of risk model in GEO training cohort and validation in TCGA-STAD cohort. (**A**) Distribution of risk scores, survival status, and gene expression heatmap of the 11 genes for the GEO training cohort. (**B**) KM plot of OS in high- and low-risk groups for GEO training cohort. (**C**) The boxplot presents the differences in risk scores among the three clusters. (**D**,**E**) Distribution of risk scores, survival status, gene expression heatmap, and Kaplan–Meier curve of OS in high- and low-risk groups for TCGA-STAD validation cohort. GEO, Gene Expression Omnibus; KM, Kaplan–Meier; OS, overall survival; TCGA-STAD, The Cancer Genome Atlas-stomach adenocarcinoma.

We next employed prognosis-related DEGs for Least Absolute Shrinkage and Selection Operator (LASSO) regression followed by multivariate Cox regression analysis (Figure S3A,B). The risk score for each patient was computed as the following formula: *CALU* × 0.13407 + *FGF1* × 0.13931 − *IRF8* × 0.09859 − *PKP2* × 0.13472 + *RBP1* × 0.12649 + *SLC39A13* × 0.27248 − *SMARCD3* × 0.01706 + *SVBP* × 0.24733 + *SWI5* × 0.35781 + *VAT1* × 0.16939 + *VWA1* × 0.28706 (Figure S3C). Patients in the combined cohorts were stratified into high- and low-risk subgroups based on the median cutoff score. KM survival analysis revealed a notably worse OS for high-risk subgroups than low-risk patients (log-rank *p* < 0.0001, Figure 2A,B). Additionally, subjects in Cluster 2 had higher risk scores (Figure 2C). To verify the repeatability and robustness of the 11-gene risk score model, we took The Cancer Genome Atlas-stomach adenocarcinoma (TCGA-STAD) (n = 144) as an external validation cohort for KM survival analysis and yielded a similar outcome (log-rank *p* = 0.022, Figure 2D,E). To explore the clinical value of the risk model, we performed univariate and multivariate Cox regression analysis. We revealed that the risk score stratification was independently associated with OS in the GEO discovery cohort and the TCGA-STAD validation cohort for AGC (Table S1). In summary, the 11-gene signature effectively predicted OS in AGC patients and showed consistent performance across cohorts.

### 2.3. Immune Landscape and Predictive Value of the Risk Model for Immunotherapy Response

**Figure 3:**
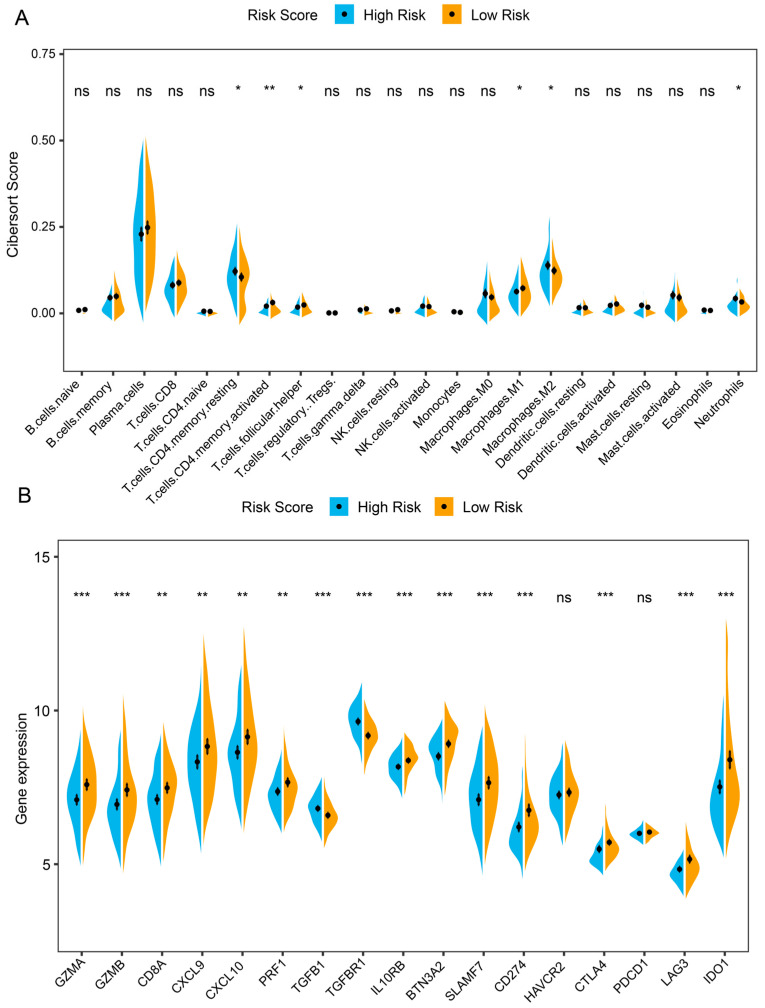
Different immune landscapes between high- and low-risk groups for GEO training cohort. (**A**) Comparison of immune cell infiltration between high- and low-risk groups using CIBERSORT analysis. (**B**) Different expression of immune-related genes between high- and low-risk groups. *p* values are marked as: ns, not significant; * *p* < 0.05; ** *p* < 0.01; *** *p* < 0.001. GEO, Gene Expression Omnibus.

Considering the impact of the tumor microenvironment on cancer prognosis, we assessed the different immune cell infiltrations between high- and low-risk groups. Compared with patients in low-risk groups, high-risk scores were accompanied by significantly reduced abundances of immune active cells, such as M1-like TAMs (Figure 3A). We also compared the expression of immune-related genes between high-risk and low-risk patients. Immune checkpoint genes were significantly up-regulated in low-risk groups, including *CD274*, *CD8A*, *LAG3*, *IDO1*, and *CTLA4* (Figure 3B). Additionally, the analysis of the TCGA-STAD cohort led to identical results (Figure S4A,B). Collectively, there was a substantial difference in the immune landscape between the high- and low-risk groups.

**Figure 4:**
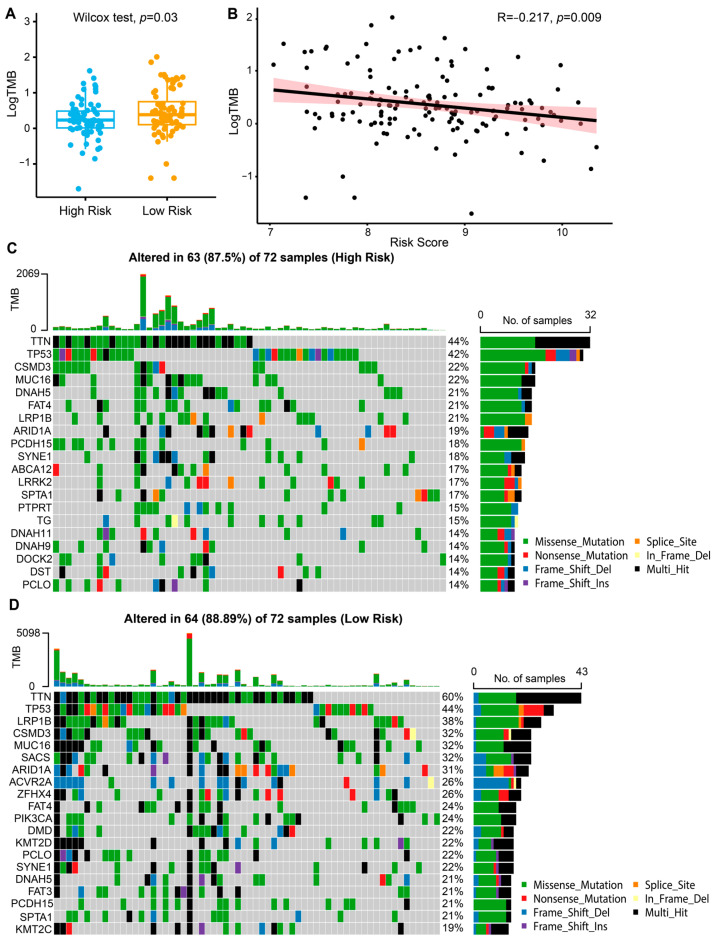
Mutational landscape analysis of risk model in TCGA-STAD cohort. (**A**) Comparison of TMB between high-risk and low-risk groups. (**B**) Pearson correlation analysis between the risk score and TMB. The red shaded area represents the 95% confidence interval around the regression line. (**C**,**D**) Waterfall plots presenting the top 20 genes with the highest mutation frequency in high-risk (**C**) and low-risk (**D**) groups. Each column represents an individual patient. The bar chart at the top displays the TMB for each patient, while the numbers on the right indicate the mutation frequency for each gene. The stacked bar chart on the right shows the distribution of mutation types for each gene, including missense mutations, nonsense mutations, frame-shift insertions/deletions, and others. TCGA-STAD, The Cancer Genome Atlas-stomach adenocarcinoma; TMB, tumor mutation burden.

Previous studies demonstrated that higher tumor mutation burden (TMB) represented a favorable outcome of immunotherapy [18]. Thus, we analyzed the correlation of TMB and risk scores in the TCGA-STAD cohort. The Wilcoxon test revealed that TMB levels were significantly lower in the high-risk group compared to the low-risk group (*p* = 0.03, Figure 4A). Pearson correlation analysis further identified a negative correlation between TMB levels and continuous risk scores (R = −0.217, *p* = 0.009, Figure 4B). To visualize the mutation landscapes among the risk subgroups, we drew waterfall plots to display the top 20 significantly mutated genes in the TCGA-STAD dataset (Figure 4C,D). The mutation frequencies of *TTN* and *TP53* were higher in both subgroups, mainly the missense mutation. In general, subjects with low-risk scores had higher frequencies of genetic mutations.

**Figure 5:**
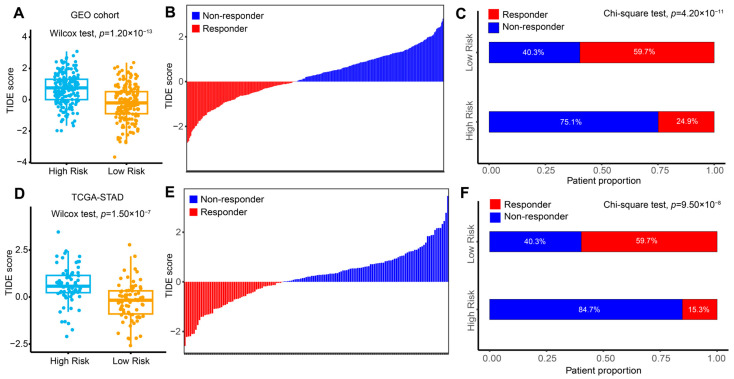
High-risk scores predict poor immunotherapy response based on TIDE analysis. (**A**,**D**) Boxplots comparing TIDE scores between high-risk and low-risk groups in the GEO cohort (**A**) and TCGA-STAD cohort (**D**) using the Wilcoxon test. (**B**,**E**) Waterfall plots showing the distribution of immunotherapy responders (red) and non-responders (blue) based on TIDE scores in the GEO cohort (**B**) and TCGA-STAD cohort (**E**). (**C**,**F**) Stacked bar charts illustrating the proportions of responders and non-responders in high-risk and low-risk groups in the GEO cohort (**C**) and TCGA-STAD cohort (**F**), analyzed by the Chi-square test. GEO, Gene Expression Omnibus; TCGA-STAD, The Cancer Genome Atlas-stomach adenocarcinoma; TIDE, Tumor Immune Dysfunction and Exclusion.

Since previous studies have demonstrated that immune profiles are correlated with cancer immunotherapy [18,19,20], we may conclude that our risk signatures could predict the immunotherapy response for AGC. Due to the lack of an available GC immunotherapy cohort for model validation, we applied the Tumor Immune Dysfunction and Exclusion (TIDE) algorithm to estimate the immune dysfunction to examine the association between the risk model and immunotherapy response in the GEO discovery and TCGA validation cohorts. In the discovery cohort, AGC patients in the low-risk group demonstrated significantly lower TIDE scores compared to those in the high-risk group (Wilcoxon test, *p* = 1.20 × 10^−13^, Figure 5A), suggesting a higher sensitivity to immunotherapy. Complementing this, the waterfall plot displays the distribution of responders and non-responders to immunotherapy based on TIDE scores (Figure 5B). The stacked bar chart further reveals that the low-risk group had a substantially higher percentage of responders (59.7%) compared to the high-risk group (24.9%) (Chi-square test, *p* = 4.20 × 10^−11^, Figure 5C). Similar findings were observed in the TCGA cohort. Patients in the low-risk group exhibited significantly lower TIDE scores (Wilcoxon test, *p* = 1.50 × 10^−7^, Figure 5D), a comparable distribution pattern of TIDE scores among responders and non-responders (Figure 5E), and a higher proportion of responders (59.7% vs. 15.3%) (Chi-square test, *p* = 9.50 × 10^−8^, Figure 5F). These results indicate that the risk model may serve as a reliable predictor of cancer immune escape and immunotherapy response, supporting our initial hypothesis.

### 2.4. Identifying IRF8 as a Hub Gene in the Risk Signature and Further Investigating Its Role in Immune Activation

**Figure 6:**
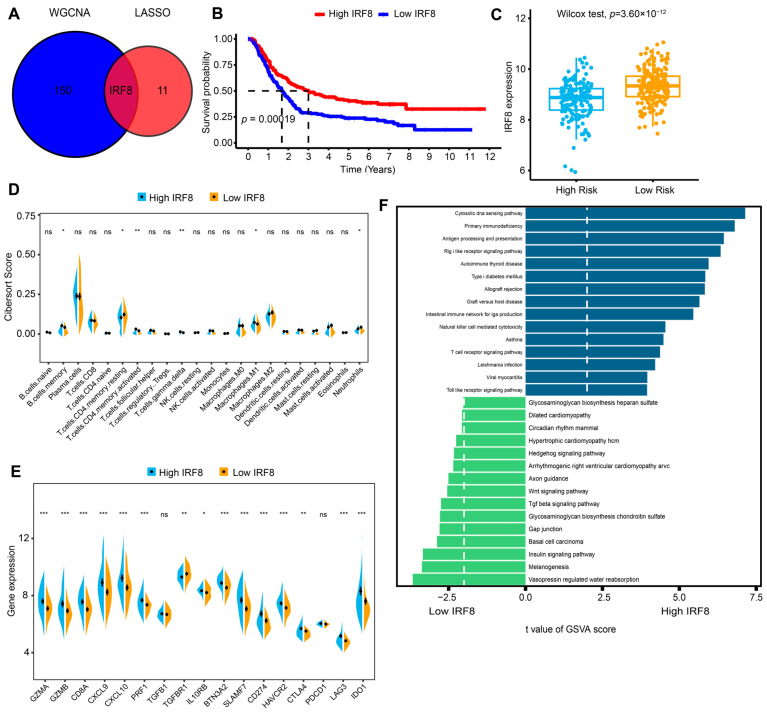
Identifying *IRF8* as a hub gene and its impact on AGC prognosis. (**A**) Venn plot showing the intersection of the hub gene of M1-TAM-related WGCNA and LASSO module. (**B**) KM survival analysis for high and low *IRF8* expression in the GEO cohort. (**C**) The difference between high and low *IRF8* expression from the GEO cohort through the Wilcox test. (**D**,**E**) Comparison of immune cell infiltration and immune-related genes between high and low *IRF8* expression. (**F**) GSVA analysis revealed the top 15 significantly enriched KEGG pathways for high and low *IRF8* expression. *p* values are marked as ns, not significant; * *p* < 0.05; ** *p* < 0.01; *** *p* < 0.001. AGC, advanced gastric cancer; GEO, Gene Expression Omnibus; GSVA, Gene set variation analysis; *IRF8*, Interferon regulatory factor 8; KEGG, Kyoto Encyclopedia of Genes and Genomes; KM, Kaplan–Meier; LASSO, Least Absolute Shrinkage and Selection Operator; TAMs, tumor-associated macrophages; WGCNA, weighted correlation network analysis.

To explore the hub gene among the M1-like TAM-related prognostic risk signature, we overlapped the 11-gene model with the genes in the magenta module. Next, *IRF8* was selected as a hub gene for further analysis and experimental verification (Figure 6A). KM survival analysis found that *IRF8* expression level was positively associated with the OS of AGC in the discovery cohort (log-rank *p* = 0.00019, Figure 6B). Consistently, patients with higher risk scores had significantly lower expression of *IRF8* (Figure 6C).

With the aim of investigating the underlying mechanism responsible for significantly different prognoses, the relationship between immune infiltration and *IRF8* expression was evaluated. Correlation analysis revealed that patients with high *IRF8* expression had markedly immune-active TIME and upregulated immune checkpoint genes (Figure 6D,E). In addition, Gene set variation analysis (GSVA) found that several immune-related pathways, including antigen processing and presentation and the T cell receptor signaling pathway, were markedly upregulated in the high-*IRF8* group. However, TGF-β and Wnt signaling was down-regulated (Figure 6F). These findings suggested that *IRF8* might promote a better prognosis by regulating the activation of immune-related pathways and immune-active cells.

### 2.5. IRF8 Overexpression Suppresses GC Progression In Vivo

**Figure 7:**
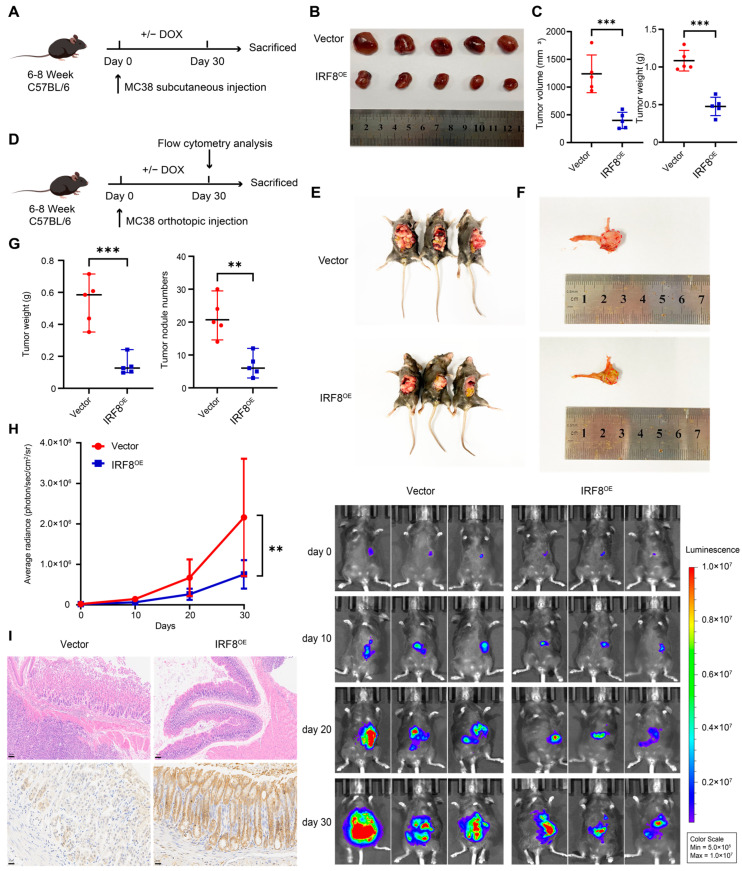
*IRF8* overexpression inhibits tumor progression in different GC models. (**A**) Schematic of the GC subcutaneous tumor model. C57BL/6 mice were subcutaneously injected with MC38 cells (5 × 10^6^ cells per mouse) and then treated with or without doxycycline. (**B**,**C**) Tumor, final tumor volume, and tumor weight in C57BL/6 mice bearing *IRF8* overexpression or vector MC38 cells. (**D**) Schematic of the GC orthotopic tumor model. MC38 cells were injected into the stomach wall (10^6^ cells per mouse) and then treated with or without doxycycline. At the experimental endpoint, mice were sacrificed to harvest tumors and spleens for flow cytometric analysis. (**E**,**F**) Representative images of abdominal metastatic tumor nodules and orthotopic tumors of orthotopic GC models in each group. (**G**) Orthotopic tumor weight and the number of GC models. (**H**) Tumors were imaged every 10 days using bioluminescence imaging to determine tumor growth and invasion. Representative images are shown on the right. (**I**) Representative images of IHC staining of *IRF8* in orthotopic tumors. Data are presented as mean ± s.d. Statistical analysis was performed using two-sided Student’s *t*-test for comparisons in (**C**,**G**) and two-way ANOVA in (**H**). ** *p* < 0.01, *** *p* < 0.001; n = 5 per group. DOX, doxycycline; GC, gastric cancer; IHC, Immunohistochemistry; *IRF8*, Interferon regulatory factor 8; OE, overexpression.

On the basis of the correlation between *IRF8* expression and GC outcomes, we next considered the impact of *IRF8* expression on tumor growth in different GC mouse models. First, we established immune-competent GC models by inoculating MC38 subcutaneously (Figure 7A). After 30 days, both tumor volume and tumor weight were reduced in the dox-induced *IRF8* overexpression group compared to the vector (Figure 7B,C). To simulate the effect of a specific GC TIME on tumor growth, we also constructed MC38-challenged orthotopic GC models on C57BL/6 mice (Figure 7D). This indicated that *IRF8* overexpression dramatically inhibited tumor progression and metastatic nodule numbers (Figure 7E–H). We performed Immunohistochemistry (IHC) analysis on gastric orthotopic tumors to confirm the efficiency of overexpression (Figure 7I). Collectively, these demonstrated the protective role of *IRF8* on GC growth and metastasis.

### 2.6. IRF8 Reshapes the Suppressive TIME in GC

**Figure 8:**
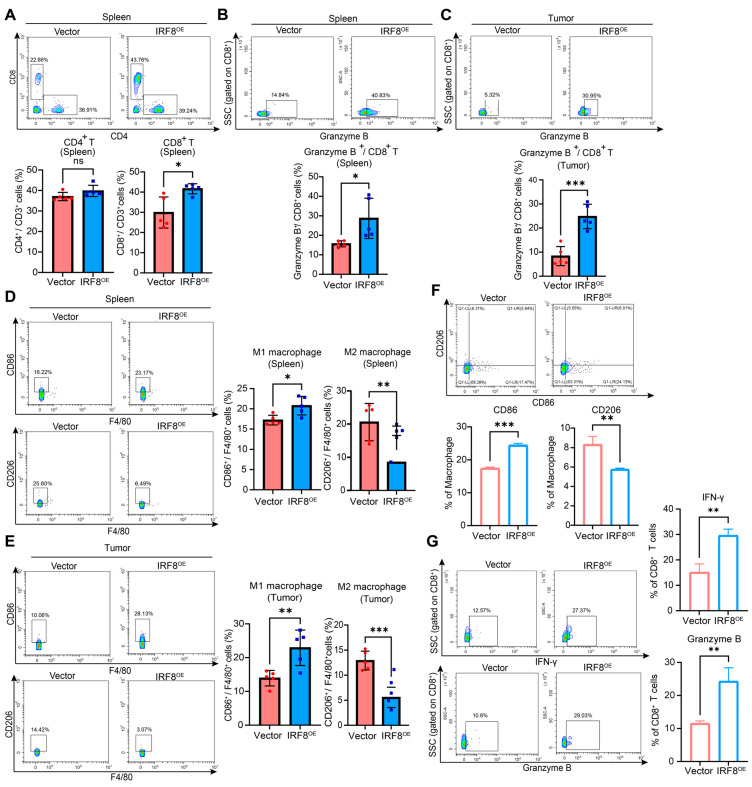
*IRF8* overexpression reshapes the immune-suppressive TIME in vivo and in vitro. Percentages of (**A**) CD3^+^CD4^+^ T cells and CD3^+^CD8^+^ T cells, (**B**,**C**) Granzyme B^+^/CD8^+^ T cells, and (**D**,**E**) CD86^+^/F4/80^+^ cells and CD206^+^/F4/80^+^ cells in tumors and spleens of the orthotopic GC models were measured by flow cytometry at the experimental endpoint (n = 5 per group). (**F**) In vitro, after being treated with the CM of *IRF8*-overexpressed or empty vector MKN45, the polarization of THP-1 was detected through flow cytometry analysis. (**G**) For IFN-γ and Granzyme B, α-CD3/CD28-pretreated CD3^+^T cells were co-cultured with TAMs induced by CM from *IRF8*-overexpressed or control samples in vitro. Cells were collected to perform flow cytometry analysis (n = 3 per group). All data are presented as mean ± s.d. Two-sided Student’s *t*-test was used for statistical analysis. ns, not significant; * *p* < 0.05; ** *p* < 0.01; *** *p* < 0.001. GC, gastric cancer; *IRF8*, Interferon regulatory factor 8; OE, overexpression; TIME, tumor immune microenvironment.

To describe the TIME of GC, we further performed flow cytometry analysis of the tumor and spleen in orthotopic GC models with dox or control treatment. The results showed that *IRF8* overexpression had an increased proportion of CD8^+^ T cells and Granzyme B^+^ CD8^+^ T cells (Figure 8A–C). In addition, *IRF8* overexpression groups had a higher tumoral and splenic infiltration of the M1 macrophage, whereas the M2 macrophage was decreased (Figure 8D,E). IHC analysis of gastric orthotopic tumors further supported the above results (Figure S5).

Based on the important impact of *IRF8* on TIME remodeling, we additionally assessed whether *IRF8* could regulate macrophage polarization and T cell cytotoxicity in vitro using human THP1 and T cells. Consistent with the results of flow cytometry in mouse models, *IRF8* could induce increased percentages of CD86^+^ TAMs but decreased CD206^+^ TAMs in THP1 (Figure 8F). In addition, we co-cultured the α-CD3/CD28-pretreated CD3^+^ T cells with TAMs induced by the conditioned medium (CM). It was revealed that high *IRF8* overexpression could enhance the generation of IFN-γ and Granzyme B in CD8^+^ T cells (Figure 8G). Together, these findings were similar to bioinformatic analysis and demonstrated that *IRF8* could enhance M1-TAM-mediated T cell activation, which might contribute to a favorable prognosis.

### 2.7. IRF8 Overexpression Augments PD-1 Blockade in Syngeneic GC Models

**Figure 9:**
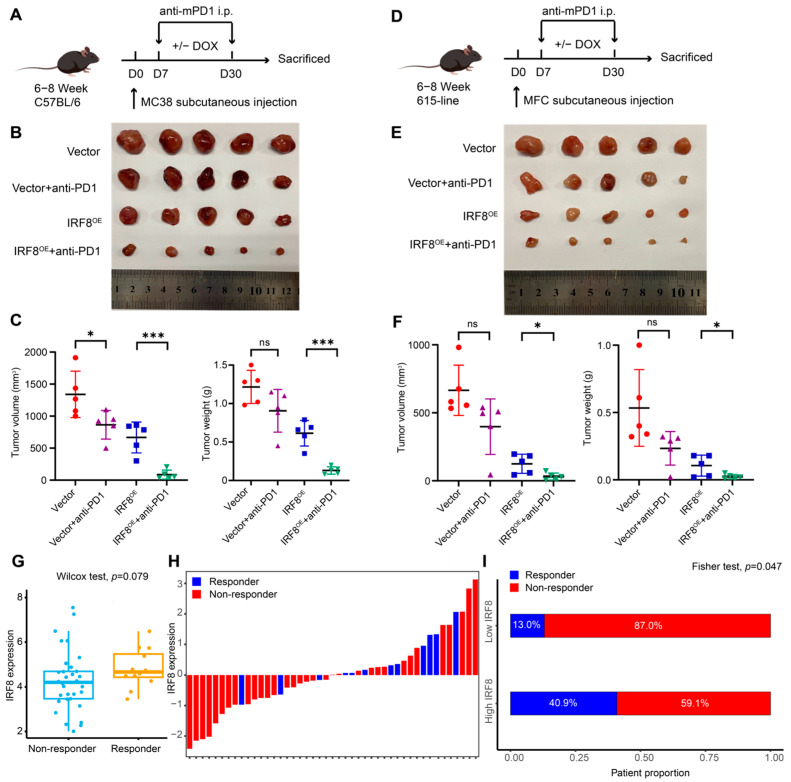
*IRF8* overexpression enhances the efficacy of anti-PD1 therapy. (**A**) Schematic of GC subcutaneous tumor model establishment and treatment diagram. C57BL/6 mice were subcutaneously injected with MC38 cells (5 × 10^6^ cells per mouse) and then treated with or without doxycycline. Anti-PD1 was delivered intraperitoneally (i.p.) (5 mg/kg) on day 7 after tumor inoculation. (**B**,**C**) Tumor, final tumor volume, and tumor weight in C57BL/6 mice were measured at the experimental endpoint. (**D**) Schematic of GC subcutaneous tumor model establishment and treatment diagram. A total of 615 mice were subcutaneously injected with MFC cells (5 × 10^6^ cells per mouse) and then treated with or without doxycycline. Anti-PD1 was delivered intraperitoneally (i.p.) (5 mg/kg) on day 7 after tumor inoculation. (**E**,**F**) Tumor, final tumor volume, and tumor weight in 615 mice were measured at the experimental endpoint. All data are presented as mean ± s.d. Two-sided Student’s *t*-test was used for statistical analysis. ns, not significant; * *p* < 0.05; *** *p* < 0.001; n = 5 per group. (**G**,**H**) Boxplot and Waterfall plot showing the different *IRF8* expression between responders and non-responders from RPJEB25780. (**I**) Bar plot showing the proportion of responders and non-responders in distinct *IRF8* expression subgroups. DOX, doxycycline; GC, gastric cancer; i.p., intraperitoneally; *IRF8*, Interferon regulatory factor 8; OE, overexpression.

To determine whether *IRF8* overexpression could improve the efficacy of anti-PD-1 therapy, 6-week-old female mice were injected with MC38 or MFC cells and treated with anti-PD-1 or controls intraperitoneally (Figure 7D and Figure 9A). The results showed that tumor growth from the mice bearing *IRF8*-overexpressing cells was significantly inhibited after anti-PD-1 treatment (Figure 9B,C). Similar results were observed in MFC-bearing mice (Figure 9E,F). Together, our findings illustrated that *IRF8* overexpression could amplify the response of anti-PD-1 treatment.

### 2.8. High IRF8 Expression Exhibits a Favorable Response to Anti-PD-1 Therapy

In light of the results of animal models, we speculated whether *IRF8* could act as a predictor of immunotherapy in AGC. Compared with the non-responders from RPJEB25780, an AGC cohort with available anti-PD1 efficacy, the responders tended to have a higher expression of *IRF8* (Figure 9G,H). Meanwhile, a higher proportion of non-responders was observed in the subgroups with low *IRF8* expression (Figure 9I). Therefore, *IRF8* may be an effective biomarker to predict clinical outcomes and anti-PD1 efficacy for AGC patients.

## 3. Discussion

Numerous studies concentrate on developing predictive models for cancer prognosis [21,22,23]. However, the high heterogeneity of GC [24] demands models that integrate multiple factors to improve prognostic accuracy and guide therapeutic strategies. Recent research has highlighted immune dysfunction as a hallmark of GC, which limits the effectiveness of immunotherapy [25,26]. Thus, exploring effective strategies for prognostic classification and discovering predictive biomarkers for AGC is necessary. In this study, we introduced a novel and robust M1-like TAM-associated risk model to predict immunotherapy outcomes in AGC. Additionally, we identified *IRF8* as a key biomarker linked to treatment efficacy. These findings offer valuable insights into enhancing the effectiveness of immunotherapy and refining personalized treatment strategies for AGC.

Substantial evidence indicates that TAMs play a pivotal role in cancer immunity and significantly influence the effectiveness of immune checkpoint blockade therapy [27,28,29]. Preclinical studies in GC have shown that reprogramming TAMs into an anti-tumorigenic phenotype can substantially enhance antitumor immunity and improve responses to immunotherapy [30,31,32,33]. Therefore, understanding the phenotypic characteristics of TAMs in GC is crucial for developing strategies to increase patients’ responsiveness to immunotherapy.

Currently, TAM-related predictive models for immunotherapy outcomes in AGC remain scarce. Gao et al. developed an M2-like TAM-related model to predict immunotherapy efficacy in AGC with ovarian metastasis [34]. However, M1- and M2-like TAMs represent two distinct polarization states that play opposing roles in the TIME, with the M1 phenotype typically associated with anti-tumor responses [35]. To date, no predictive models specifically focusing on M1-like TAMs for immunotherapy outcomes in AGC have been reported. Our risk model successfully stratified AGC patients into high- and low-risk groups. Patients in the low-risk group demonstrated improved prognoses and greater sensitivity to immunotherapy. Additionally, univariate and multivariate analyses validated the risk model as an independent prognostic predictor for AGC. By constructing an M1-like TAM-related predictive model, this study fills a critical gap, offering a novel tool for assessing immunotherapy responses in AGC and deepening our understanding of TAM polarization and its role in immune activation.

Moreover, our study recognized *IRF8* as the hub gene for further exploration. *IRF8* is a kind of interferon regulatory transcription factor, playing a crucial role in tumorigenesis, tumor development, and immune responses [36,37,38]. It has been recognized as a potential biomarker for clinical outcomes across various cancer types [39,40,41]. However, the biological function of *IRF8* in GC immunity is still unclear.

Our study revealed a positive relationship between *IRF8* expression and favorable clinical outcomes and immunotherapy response in AGC cohorts. To further explore the role of *IRF8* in regulating the TIME of GC, we conducted in vivo and in vitro experiments. The findings demonstrated that *IRF8* overexpression promoted an immune-active TIME and suppressed tumor progression. Notably, *IRF8* overexpression also enhanced the efficacy of anti-PD-1 therapy. These results are consistent with previous studies in hepatocellular carcinoma, which similarly highlighted the role of *IRF8* in fostering an immune-supportive microenvironment [40].

Mechanistically, our GSVA analysis revealed that Wnt/β-catenin and TGF-β signaling pathways were highly activated in groups with low *IRF8* expression, suggesting potential mechanisms underlying its role. Previous studies have shown that these pathways contribute to the development of an immunosuppressive TIME and resistance to immunotherapy [42,43,44,45]. Notably, inhibition of Wnt/β-catenin or TGF-β signaling in combination with anti-PD-1/PD-L1 therapies has been shown to effectively suppress tumor growth [46,47,48]. Based on these findings, we speculate that *IRF8* enhances cancer immunity and boosts the efficacy of anti-PD-1 therapy in GC by regulating Wnt/β-catenin and TGF-β signaling pathways.

A series of studies has investigated the regulatory roles of IRF family members in pathways that suppress tumor malignancy. Chen et al. reported that *IRF2* promotes *AMER1* transcription in GC, resulting in β-catenin degradation and pathway inactivation [49]. In another study, Tian et al. showed that *IRF3* inhibits β-catenin activation by preventing its nuclear translocation in colorectal cancer [50]. Additionally, research has demonstrated that *IRF8* deficiency, coupled with activated β-catenin, contributes to the progression and drug resistance of chronic myeloid leukemia [51]. Despite these advances, the mechanisms by which *IRF8* regulates β-catenin signaling to enhance the TIME in GC remain poorly understood.

We then turned our focus to the impact of IRF family members on the TGF-β signaling pathway. Studies have shown that activated *IRF3* suppresses TGF-β signaling by inhibiting *Smad3* activation and disrupting *Smad3* transcriptional complexes. This regulation affects critical processes, including cell proliferation, epithelial-mesenchymal transition, and Treg cell differentiation [52]. However, the role of *IRF8* in regulating the TGF-β signaling pathway and its influence on the immune microenvironment remains unexplored, warranting further investigation.

Our study has several limitations. First, the comprehensive assessments primarily relied on bioinformatics analyses, which require validation through multicenter studies to ensure the robustness of the findings. Second, while we conducted in vitro and in vivo experiments to demonstrate the role of *IRF8* in GC immunity and identified it as a predictor of immunotherapy response in AGC for the first time, the underlying mechanisms remain incompletely understood. Specifically, further investigation is needed to elucidate how *IRF8* regulates TAM-mediated CD8^+^ T cell activation via the Wnt/β-catenin and TGF-β signaling pathways.

Future research should aim to validate the predictive value of *IRF8* in multicenter cohorts and investigate its regulatory effects on Wnt/β-catenin and TGF-β signaling within the TIME. These efforts will enhance our understanding of *IRF8*′s role in AGC and guide the development of more effective immunotherapy strategies.

## 4. Materials and Methods

### 4.1. Data Collection and Preprocessing

A total of 362 AGC samples were obtained from the GEO database (GSE66229, GSE57303, and GSE15459). These datasets were combined using the “ComBat” algorithm [53] and served as the discovery cohort. Samples lacking RNA sequencing data and complete survival information were excluded. The TCGA-STAD dataset with 144 AGC was treated as an external validation cohort. To assess the correlation between *IRF8* expression and response to immunotherapy, we analyzed the RPJEB25780 AGC cohort, which includes data on anti-PD1 treatment efficacy.

### 4.2. Analysis of Tumor-Infiltrating Immune Cells

We applied the CIBERSORT package [54] to calculate the relative abundance of 22 immune cells in the TIME. Using the median level of infiltration of each immune cell type as a cut-off, patients were classified into either high- or low-infiltration groups.

### 4.3. WGCNA

We used the WGCNA algorithm [55] to recognize the gene module highly related to M1-like TAM infiltration. After filtering out low-expression genes, the expression matrix from the discovery cohort was used for WGCNA. Sample clustering was conducted to exclude outliers. The PickSoftThreshold function in R was employed to determine the minimum soft threshold power (β) with R^2^ > 0.9 to achieve scale-free topology. Subsequently, the topological overlap matrix and its dissimilarity were computed to generate a hierarchical clustering tree. The most relevant gene module was identified using Pearson correlation coefficients and corresponding *p*-values.

### 4.4. Identification of M1-like TAM-Related Subgroups via Consensus Clustering

Genes from the module most strongly correlated with M1-like TAM infiltration were further analyzed for their association with OS in AGC. A total of 36 survival-associated genes were selected and subjected to the ConsensusClusterPlus R package [56], with 1000 iterations to ensure stable classification. The optimal number of clusters (K value) was determined using the Consensus Cumulative Distribution Function (CDF) plot.

### 4.5. Construction and Validation of M1-like TAM-Related Prognostic Risk Model

The limma package [57] was applied to determine the DEGs among the three clusters. Subsequently, univariate Cox regression analysis was used to select DEGs significantly associated with OS. To refine the candidate genes, we used LASSO regression with 10-fold cross-validation, followed by a multivariate proportional hazards model [58]. The risk score was calculated using the following formula: Risk score = ∑Coef Genes × Exp Genes. Based on the median risk score, patients were stratified into high-risk and low-risk groups. Survival differences between these groups were analyzed using the survival and survminer R packages, while the distribution of OS time and status was visualized with ggrisk. Finally, univariate and multivariate Cox regression analyses were conducted while integrating the risk model with clinical parameters.

### 4.6. Functional Enrichment Analysis

To explore pathway enrichment, gene sets for GO and KEGG were sourced from the Molecular Signatures Database (https://www.gsea-msigdb.org/gsea/msigdb/) (accessed on 20 January 2024) [59]. GSVA [60] was performed to assess differences in pathway enrichment between the subgroups. Pathways were considered significantly enriched if both *p*-values and q-values were below 0.05.

### 4.7. Somatic Mutation Analysis

To evaluate the mutation landscape of the high- and low-risk subgroups, we calculated the TMB using the maftools R package on the TCGA-STAD dataset [61]. An oncoplot was generated to visualize the top 20 most frequently mutated genes and their mutation types.

### 4.8. Immunotherapy Response Prediction

The TIDE framework was employed to predict immune escape and response to immunotherapy based on gene expression profiles [62]. Patients with elevated TIDE scores were classified as likely non-responders to immunotherapy.

### 4.9. Cell Culture and Stable Transfection

The human GC cell line MKN45 and the human monocytic cell line THP-1 (ATCC; Manassas, VA, USA) were used for in vitro experiments, while mouse gastrointestinal tumor cell lines MC38 and MFC were employed for establishing mouse models. All cells were cultured in RPMI 1640 medium (Gibco; Thermo Fisher Scientific; Waltham, MA, USA) supplemented with 10% fetal bovine serum (FBS) at 37 °C in a 5% CO_2_ atmosphere. Lentiviral vectors carrying the pCDH plasmid for *IRF8* overexpression or an empty control vector (for both human and mouse cells) were obtained from SYNBIO Technologies (Suzhou, China). Following lentiviral transduction, cells were selected with puromycin for two weeks. Successful transfection was confirmed via Western blotting before cells were used in subsequent functional assays.

### 4.10. Preparation of CM

After adherent growth for 24 h, the culture medium of MKN45 was replaced with serum-free medium, and cells were incubated for an additional 24 h. The supernatant was collected and used as conditioned medium for macrophage polarization.

### 4.11. Generation of Human T Cells

Human peripheral blood mononuclear cells (PBMCs) were obtained from healthy donors with informed consent at San Yat-sen University Cancer Center. PBMCs were isolated from fresh blood by Ficoll lymphocyte separation solution and cultured in RPMI 1640 medium containing 10% FBS. Human naive CD3^+^ T cells were purified and pre-stimulated with anti-CD3/CD28 antibodies (STEMCELL Technologies, Vancouver, BC, Canada, #10971) for subsequent experiments.

### 4.12. Western Blotting

Cells from MKN45, MC38, and MFC lines were lysed using a protein extraction buffer. Proteins were separated on a 10% SDS-PAGE gel and transferred to a PVDF membrane. After blocking with 5% non-fat milk, membranes were incubated with primary antibodies followed by secondary antibodies for detection. The primary antibodies used were anti-*IRF8* (Affinity, Changzhou, China, 1:500, #DF13627) and anti-*GAPDH* (Cell Signaling Technology, Danvers, MA, USA, 1:1000, #2118). The overexpression of *IRF8* was verified by Western blotting (Figure S6).

### 4.13. Animal Models

To establish subcutaneous GC models, MC38 or MFC cells (5 × 10^6^) were resuspended in 100 μL phosphate buffered saline (PBS) and implanted into the hind flank of 6-week-old female C57BL/6 or 615 mice. For orthotopic GC models, MC38 cells (10^6^) were mixed with Matrigel and injected into the stomach wall of 6-week-old female C57BL/6 mice. Tumor growth was monitored every 10 days by a living imaging system. Tumor volume and tumor weight were measured at the experimental endpoint.

### 4.14. Multicolor Flow Cytometry Analysis

At the endpoint of animal experiments, orthotopic tumors and spleens were harvested for flow cytometry analysis. Cells were stained with surface or intracellular fluorescent antibodies. For in vitro experiments, human TAMs and T cells were similarly analyzed. To assess IFN-γ and Granzyme B expression, cells were incubated with a Cell Stimulation Cocktail (BD Biosciences, San Jose, CA, USA, #555028) at 37 °C with 5% CO_2_ for 4 h. Dead cells were stained using BD Horizon™ Fixable Viability Stain 510 (BD Biosciences, San Jose, CA, USA, #564406). The following antibodies were used for flow cytometry. Mouse-specific: anti-CD45 (BioLegend, San Diego, CA, USA, #103108, #103128), anti-CD11b (Invitrogen, Carlsbad, CA, USA, #17-0112-82), anti-F4/80 (Invitrogen, Carlsbad, CA, USA, #12-4801-82), anti-CD86 (Invitrogen, Carlsbad, CA, USA, #25-0862-82), anti-CD206 (Invitrogen, Carlsbad, CA, USA, #56-2061-82), anti-CD3 (BioLegend, San Diego, CA, USA, #100306), anti-CD4 (BioLegend, San Diego, CA, USA, #100548), and anti-CD8α (BioLegend, San Diego, CA, USA, #100708). Human-specific: anti-CD68 (BioLegend, San Diego, CA, USA, #333809), anti-CD86 (BioLegend, San Diego, CA, USA, #305421), anti-CD206 (BioLegend, San Diego, CA, USA, #321105), anti-CD8 (BioLegend, San Diego, CA, USA, #344723), anti-IFN-γ (BioLegend, San Diego, CA, USA, #502517), and anti-human/mouse Granzyme B (BioLegend, San Diego, CA, USA, #372203). After staining, cells were resuspended in PBS containing 2% FBS for flow cytometric analysis.

### 4.15. IHC Staining

Gastric orthotopic tumor samples from mice were fixed in 10% formalin and subsequently embedded in paraffin. The paraffin-embedded tissue blocks were sectioned into slices. After deparaffinization, rehydration, and antigen retrieval, sections were incubating with 1% bovine serum albumin in PBS for 20 min and subsequently incubated with the primary antibody overnight. Following incubation with secondary antibodies, sections were stained with diaminobenzidine substrate. The sections were then counterstained with hematoxylin, dehydrated through a series of graded alcohols, and mounted with a coverslip for imaging. The following primary antibodies were used: anti-*IRF8* (Affinity, Changzhou, China, 1:100, #DF13627), anti-CD86 (Servicebio, Wuhan, China, 1:200, #GB115630), anti-CD163 (Servicebio, Wuhan, China, 1:500, #GB15340), and anti-CD8 (Cell Signaling Technology, Danvers, MA, USA, 1:200, #98941S).

### 4.16. Statistical Analysis

KM survival analysis, along with the log-rank test, was used to evaluate differences in OS between groups. Univariate and multivariate Cox regression analyses were conducted to identify independent prognostic factors for OS. Continuous variables were compared using the Wilcoxon or Kruskal–Wallis tests, while categorical variables were analyzed using chi-square or Fisher’s exact tests. Pearson’s correlation coefficient was employed for correlation analysis. All statistical analyses were performed using R software (v4.0). Statistical significance was set to a two-tailed *p*-value of <0.05.

## 5. Conclusions

Our study established a prognostic signature based on M1-like TAMs for AGC. Further analysis of *IRF8* highlighted its role in TAM reprogramming and antitumor immunity by CD8^+^ T cells, confirming it as a potential predictive biomarker for sensitivity to immunotherapy in AGC.

## Data Availability

All the data corresponding to the STAD series used in the current study are available in GEO (https://www.ncbi.nlm.nih.gov/geo) (accessed on 20 November 2023) and TCGA (https://portal.gdc.cancer.gov/) (accessed on 10 February 2024) repositories.

## Supplementary Materials

The following supporting information can be downloaded at: https://www.mdpi.com/article/10.3390/ijms26031089/s1.

## Abbreviations

AGC: advanced gastric cancer; GC: gastric cancer; TIME: tumor immune microenvironment; TAMs: tumor-associated macrophages; *IRF8*: Interferon regulatory factor 8; GEO: Gene Expression Omnibus; TCGA: The Cancer Genome Atlas; TCGA-STAD: The Cancer Genome Atlas-stomach adenocarcinoma; OS: overall survival; KM: Kaplan–Meier; WGCNA: weighted correlation network analysis; DEGs: differentially expressed genes; GO: Gene Ontology; KEGG: Kyoto Encyclopedia of Genes and Genomes; GSVA: Gene set variation analysis; LASSO: Least Absolute Shrinkage and Selection Operator; TMB: tumor mutation burden; TIDE: Tumor Immune Dysfunction and Exclusion; CM: Conditioned Medium.
