# Supplementary material for: Signature Construction Associated with Tumor-Infiltrating Macrophages Identifies IRF8 as a Novel Biomarker for Immunotherapy in Advanced Gastric Cancer
Source: Int J Mol Sci. 2025 Jan 27;26(3):1089. doi: 10.3390/ijms26031089 (PMC11817691; doi:10.3390/ijms26031089)
Supplement: Supplementary file 1 [file ijms-26-01089-s001.zip › Supplementary Materials Figure legends.pdf]

Supplementary Materials:

Table S1. Univariate and multivariate Cox analysis of risk scores and clinical features.

Figure S1. Flow chart of data analyses.

Figure S2. WGCNA identifies an M1-like TAMs-related module. Consensus clustering defines the stable M1-like TAMs-related clusters in the GEO training cohort. (A-B) The graphs showed the scale-free fit index and mean connectivity for different soft-thresholding powers. (C) Gene dendrogram and module colors. (D-E) CDF and relative change in area under CDF curve for K = 2–6. (F) Cluster consensus value of 2 clusters. CDF, Cumulative Distribution Function; GEO, Gene Expression Omnibus; TAMs, tumor-associated macrophages; WGCNA, weighted correlation network analysis.

Figure S3. Construction of LASSO-Cox model. (A) Log Lambda value in LASSO model. (B) 10-fold cross-validation to get the most optimal parameters for LASSO regression. (C) Forest plot showing the hazard ratios of the 11 genes in the risk model. LASSO: Least Absolute Shrinkage and Selection Operator.

Figure S4. Different immune landscapes between high and low-risk groups for TCGA-STAD cohort. (A) Comparison of immune cell infiltration between high and low-risk groups using CIBERSORT analysis. (B) Different expression of immune-related genes between high and low-risk groups. P values are marked as: ns, not significant; * $p < 0.05$; ** $p < 0.01$; *** $p < 0.001$. TCGA-STAD: The Cancer Genome Atlas-stomach adenocarcinoma.

Figure S5. Representative images of IHC staining for IRF8, CD86, CD163 and CD8 in orthotopic tumors. IHC, Immunohistochemistry; *IRF8*, Interferon regulatory factor 8.

Figure S6. The overexpression of *IRF8* was verified by Western blotting. *IRF8*, Interferon regulatory factor 8; OE, overexpression.
