# Supplementary material for: Signature Construction Associated with Tumor-Infiltrating Macrophages Identifies IRF8 as a Novel Biomarker for Immunotherapy in Advanced Gastric Cancer
Source: Int J Mol Sci. 2025 Jan 27;26(3):1089. doi: 10.3390/ijms26031089 (PMC11817691; doi:10.3390/ijms26031089)
Supplement: Supplementary file 1 [file ijms-26-01089-s001.zip › Table S1.pdf]

	Univariate analysis		Multivariate analysis	
	HR (95% CI)	p value	HR (95% CI)	p value
**GEO training cohort (n = 362)**				
Stage (IV VS III)	2.584 (1.986-3.362)	<0.001	2.620 (2.010-3.415)	<0.001
Age	1.014 (1.003-1.026)	0.014	1.016 (1.004-1.027)	0.007
Sex (Female VS Male)	0.976 (0.739-1.289)	0.863		
Risk (High VS Low)	2.452 (1.873-3.209)	<0.001	2.395 (1.828-3.137)	<0.001
**TCGA-STAD validation cohort (n = 144)**				
Stage (IV VS III)	1.454 (0.807-2.621)	0.213		
Age	1.035 (1.011-1.060)	0.004	1.036 (1.012-1.061)	0.003
Sex (Female VS Male)	0.992 (0.605-1.626)	0.973		
Risk (High VS Low)	1.739 (1.075-2.811)	0.024	1.773 (1.097-2.865)	0.019

GEO, Gene Expression Omnibus; TCGA-STAD, The Cancer Genome Atlas-stomach adenocarcinoma.
