# Supplementary figures and images for: Signature Construction Associated with Tumor-Infiltrating Macrophages Identifies IRF8 as a Novel Biomarker for Immunotherapy in Advanced Gastric Cancer
Source: Int J Mol Sci. 2025 Jan 27;26(3):1089. doi: 10.3390/ijms26031089 (PMC11817691; doi:10.3390/ijms26031089)

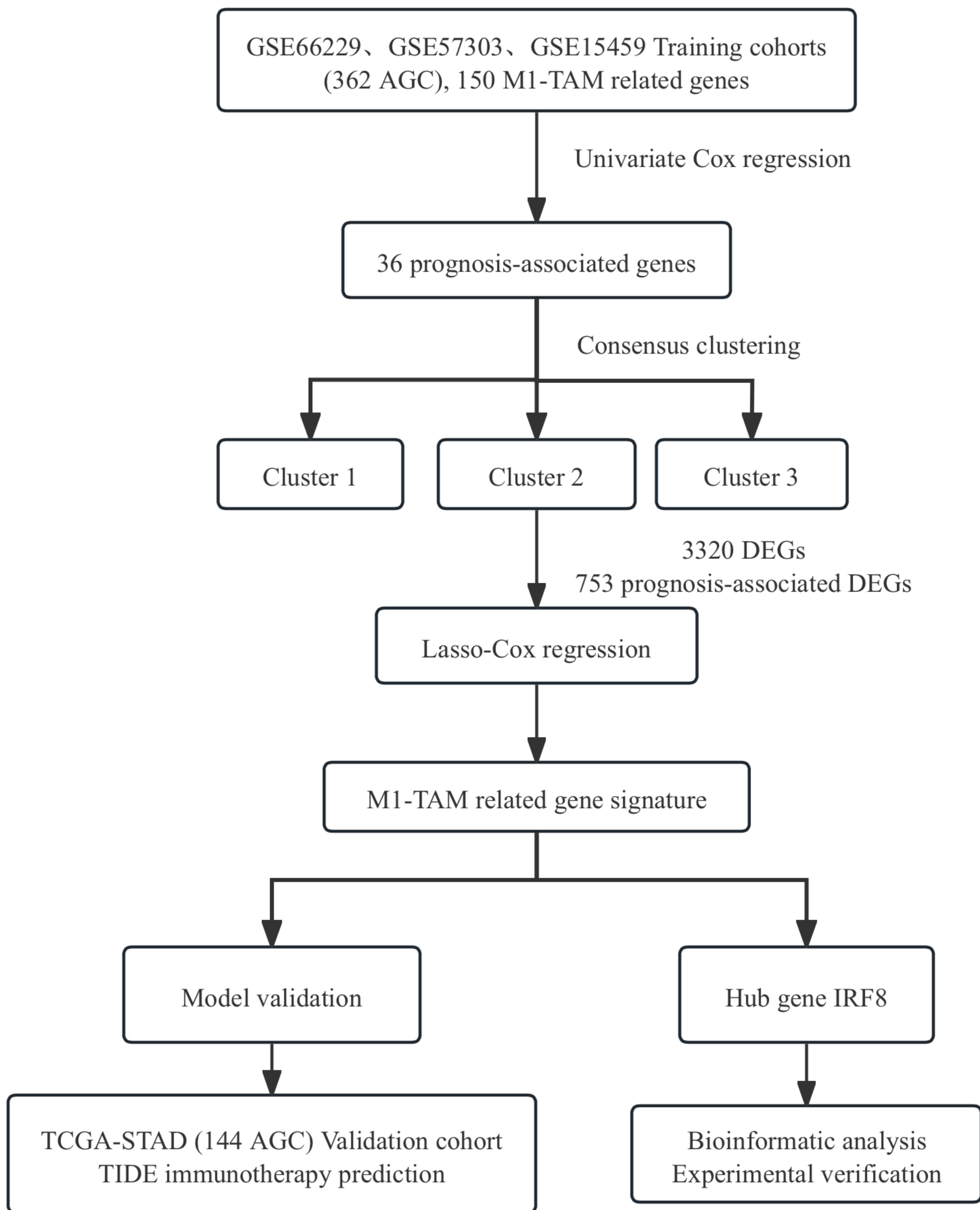

Supplement: Supplementary file 1 [file ijms-26-01089-s001.zip › Figure S1.pdf]

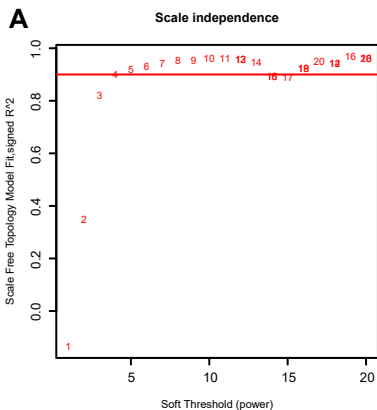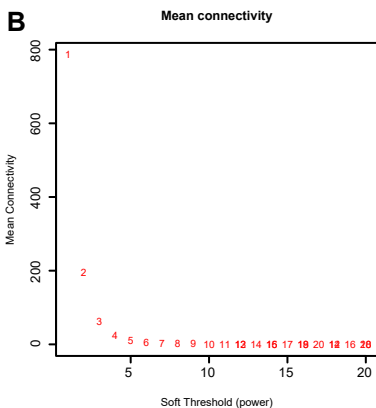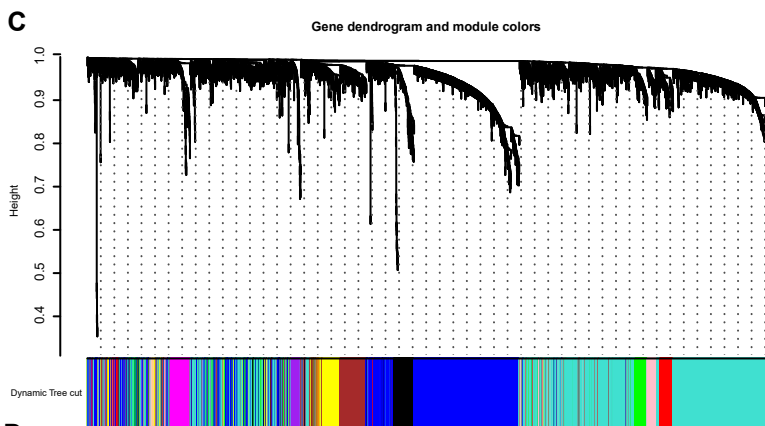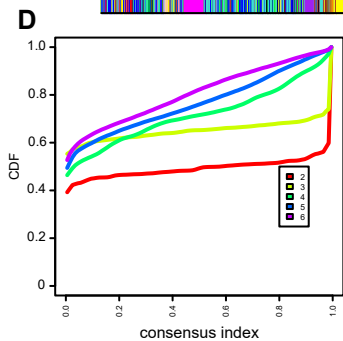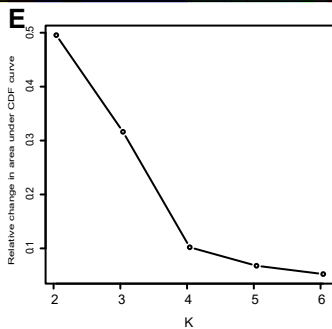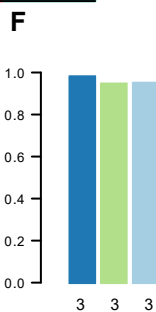

Supplement: Supplementary file 1 [file ijms-26-01089-s001.zip › Figure S2.pdf]

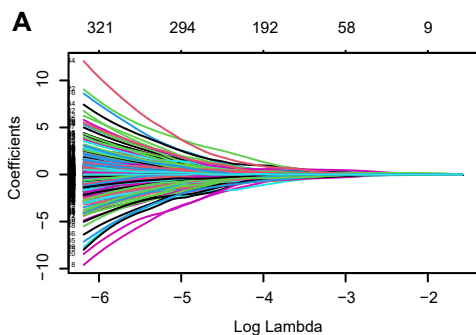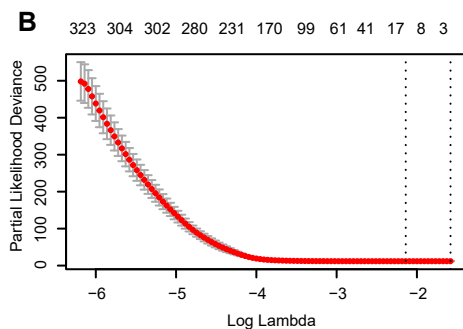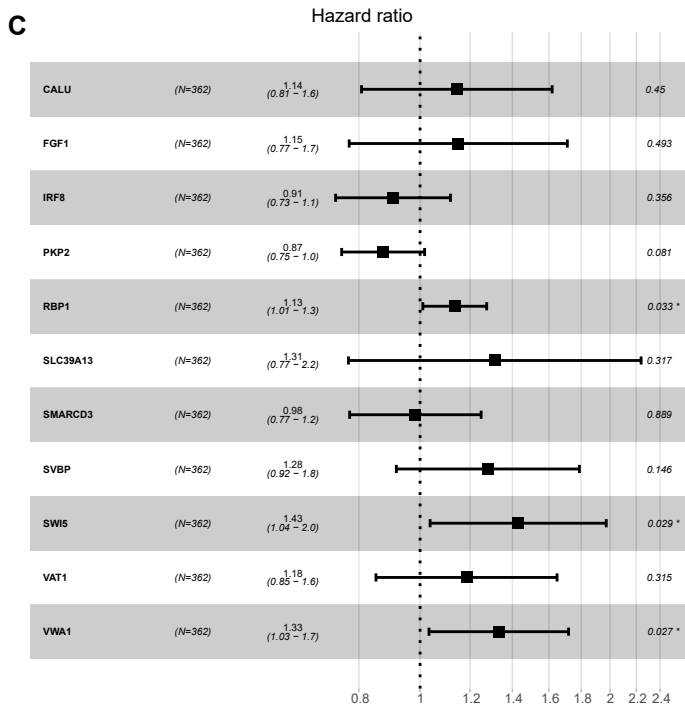

Supplement: Supplementary file 1 [file ijms-26-01089-s001.zip › Figure S3.pdf]

**A**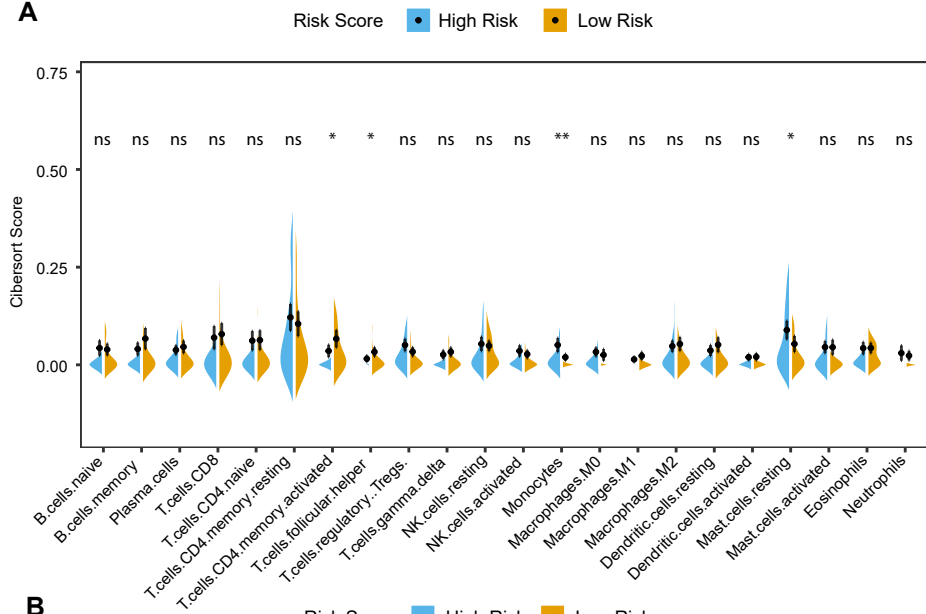**B**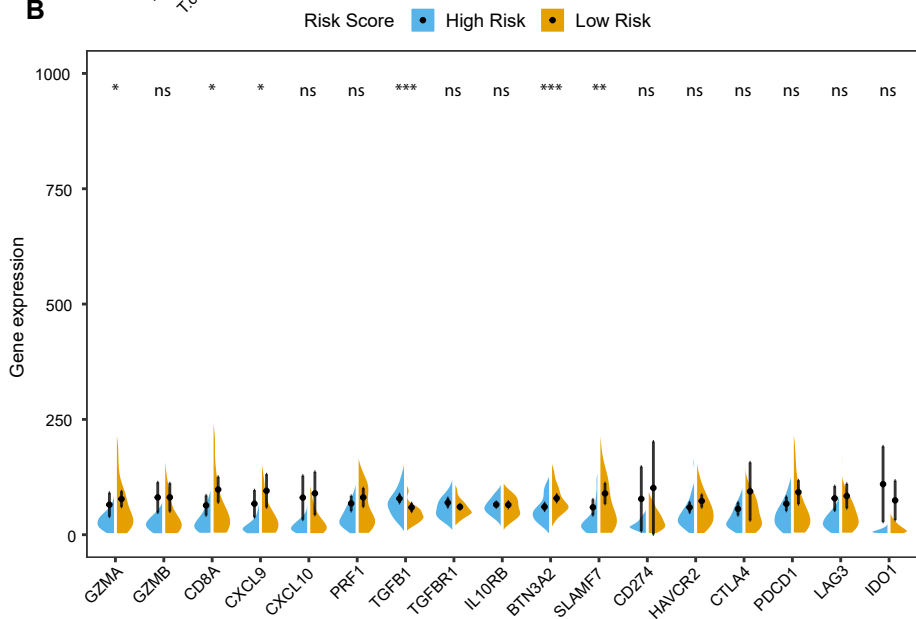

Supplement: Supplementary file 1 [file ijms-26-01089-s001.zip › Figure S4.pdf]

Vector

IRF8<sup>OE</sup>

IRF8

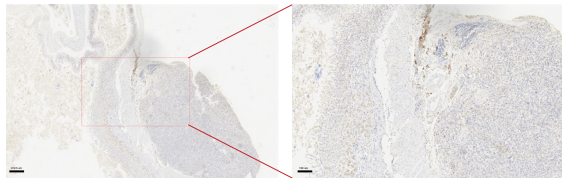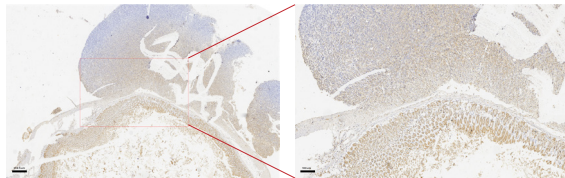

CD86

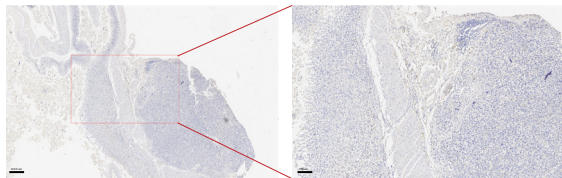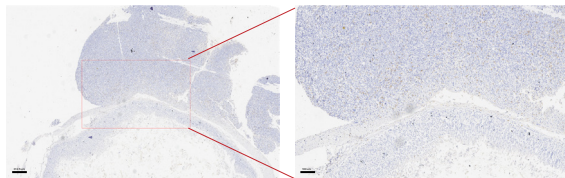

CD163

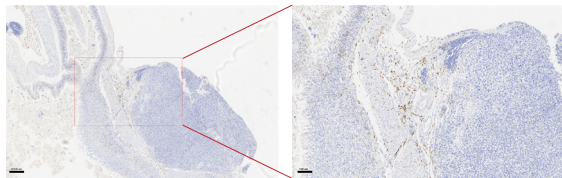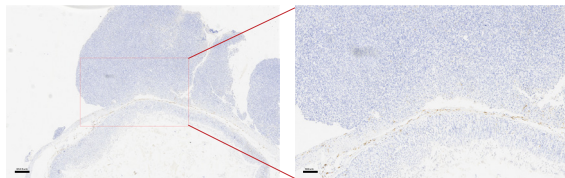

CD8

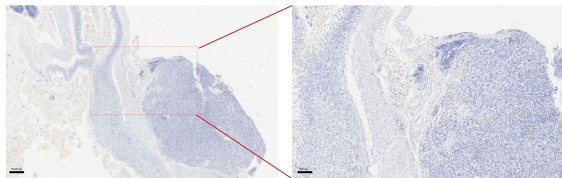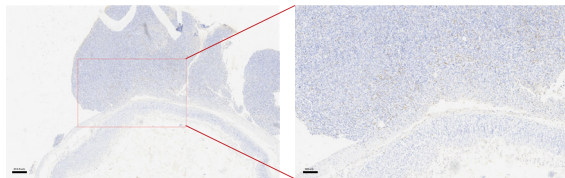

Supplement: Supplementary file 1 [file ijms-26-01089-s001.zip › Figure S5.pdf]

**A**

MKN45

IRF8

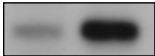

GAPDH

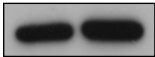Vector IRF8<sup>OE</sup>**B**

MC38

MFC

IRF8

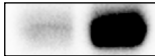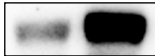

GAPDH

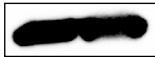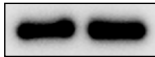Vector IRF8<sup>OE</sup>Vector IRF8<sup>OE</sup>

Supplement: Supplementary file 1 [file ijms-26-01089-s001.zip › Figure S6.pdf]
